# Isolation and antibiotic susceptibility of *Shigella* species from stool samples among hospitalized children in Abadan, Iran 

**Published:** 2014

**Authors:** Nabi Jomezadeh, Shahram Babamoradi, Enayatollah Kalantar, Hazhir Javaherizadeh

**Affiliations:** 1 *Abadan School of Medical Sciences, Abadan, Iran*; 2 *Ilam University of Medical Sciences, Ilam, Iran*; 3 *Department of Pathobiology, School of Medicine, Alborz University of Medical Sciences, Karaj, Iran*; 4 *Department of Pediatric Gastroenterology, Namazi Hospital, Shiraz University of Medical Sciences, Shiraz, Iran *

**Keywords:** Antimicrobial resistance, Children, *Shigella*

## Abstract

**Aim:** The aim of this study was to determine the incidence of *Shigella* species and their antimicrobial susceptibility patterns in hospitalized children with *Shigellosis *in Abadan, Iran.

**Background:** Shigellosis is caused by different species of *Shigella* and one of the most common causes of diarrhea in children. This disease is endemic in many developing countries including Iran.

**Patients and methods:** This prospective cross sectional study was conducted in a teaching hospital in Abadan, Iran during June 2011 to May 2013. Stool specimens were collected from pediatric age group. All isolates were confirmed as *Shigella *species by biochemical and serologic tests. Antibiotic sensitivity pattern of these isolates was studied by disk diffusion Method.

**Results:** Among all 705 stool samples, 36 (5.1%) yielded *Shigella*. Of cases, 392 (55.6%) were girl and 313 (44.4%) were boy. The most common *Shigella *isolates were *S. flexneri* (n=19, 52.7%) followed by *S. sonnei *(n=11, 30.5%), *S. boydii* (n=4, 11.1%) and *S. dysenteriae* 2(5.5%). Of the *Shigella* isolates, 47.2% showed resistance to two or more antimicrobial agents. Resistance pattern against various antimicrobials were as follows: trimethoprim-sulphamethoxazole (80.5%), ampicillin (63.8%), tetracycline (58.3%), chloramphenicol (33.3%), nalidixic acid (27.7%), and cefixime (16.6%). There was no resistance against ciprofloxacin and ceftriaxone.

**Conclusion:** The most common isolates were *S. flexneri* followed by *S. Sonnei*. There was no antibiotic resistance against ciprofloxacin and ceftriaxone. TMP-SMZ showed highest resistance pattern.

## Introduction

*Shigella *spp. belongs to the family Enterobacteriacae. It is a small, unencapsulated, non-motile gram-negative rod. There are four species of *Shigella*, classified on the basis of biochemical and serological differences: *S. dysenteriae*, *S. flexneri*, *S. boydii*, and *S. sonnei *(1). *Shigella sonnei* is found mostly in industrialized countries; *S. dysenteriae*, *S. flexneri*, and *S. boydii* are more prevalently found in developing countries. Of the estimated 164.7 million S*higella* diarrheal episodes occurring globally every year, 99% of infections occur in developing countries and the majority of cases and deaths occur amongst children less than five years of age. *Shigella* transmission is by the fecal-oral route, including direct person-to-person contact and may be indirect through ingestion of contaminated food or water. It is most likely to occur in children and those who neglect to clean hands thoroughly, including under fingernails after defecation. In as much as *Shigella* spp. have obtained multiple antimicrobial resistances, the challenge for clinical management is distinguishing which drugs preserve their activity and clinical efficacy. The Centers for Disease Control and Prevention (CDC) have suggested that sensitivity testing be accomplished to instruct selection of proper antimicrobial therapy for *Shigellosis*. Because antimicrobial susceptibility patterns of *Shigella* may differ greatly in different geographical regions and over time, supervising resistance patterns is necessary to guide selection of appropriate empirical antibiotic treatment (2-4). 

In the study by Esmaeili Dooki et al. in north of Iran, *Shigella* is the 2^nd^ most common cause of bloody and non-bloody diarrhea among bacterial gastroenteritis (5). In their study, all isolates of *Shigella* were resistant to cefixime (5). The study by Pourakbari et al., the rate of sensitivity to ceftriaxone was 95% (6). Therefore, antimicrobial resistance pattern of *Shigella* infections in infants and children is not adequately defined in this area. The aim of this study was to find frequency of shigellosis in children with diarrhea and antimicrobial resistance pattern among isolates. 

## Patients and Methods

This prospective cross sectional study was conducted in Taleghani Teaching Hospital, Abadan, Iran during June 2011 to May 2013. Seven hundred and five (n=705) stool samples were collected in clean open-mouth disposable containers from children who were clinically diagnosed as suffering from dysentery. All the samples were immediately sent to the laboratory for isolation and identification of *Shigella* organisms according to standard methods. Three different media and an enrichment medium were used for optimal isolation. The stool samples were primarily inoculated on MacConkey agar, xylose-lysine deoxycholate (XLD) agar, and Salmonella-Shigella (SS) agar. Enrichment was done in selenite F broth and incubated at 37°C for 6 hours. Subculture was done in the MacConkey agar, XLD agar and SS agar. Further incubation was done aerobically at 35-37°C for 18-24 hours. Subsequently, biochemical tests were used to confirm the bacteria including growth on TSI agar, SIM, Simmons citrate, and MR-VP reaction and etc. Identification and serotyping of *Shigella* were performed by doing slide agglutination test using *Shigella* polyvalent antiser (Denka Seiken Co. Ltd, Tokyo, Japan). Antimicrobial susceptibility of *Shigella* strains was determined by the disc diffusion method in accord with the guidelines of the Clinical Laboratory Standards Institute (CLSI). The antibiotics used were ampicillin (10μg), tetracycline (30μg), trimethoprim-sulphamethoxazole (1.25/23.75μg), nalidixic acid (30mg), ceftriaxone (30μg), chloramphenicol (30μg), ciprofloxacin (5μg), cefixime (5μg), and gentamicin (10μg) (Oxoid, UK). *E. coli* ATCC 25922 strain was used as quality control for susceptibility tests. 

**Table 1:** Distribution of the age groups of children according to *Shigella* species

**Age group** **(years)**	***S. flexneri*** **n (%)**	***S. sonnei*** **n (%)**	**n (%)**	***S. dysenteriae***	**Total** **n (%)**
<1	2(10.5)	1(9)	0	0	3(8.3)
1-5	9(47.3)	6(54.5)	3(75)	2(100)	20(55.5)
6-11	5(26.3)	2(18.1)	1(25)	0	8(22.2)
12-15	3(15.7)	2(18.1)	0	0	5(13.8)
Total	19(52.7)	11(30.5)	4(11.1)	2(5.5)	36(100)

**Table 2:** Antibiotic resistance patterns of the *Shigella* isolates according to the *Shigella* species

**Antibiotic**	***S. flexneri*** **n= 19 (%)**	***S. sonnei*** **n= 11(%)**	**n= 4 (%)**	***S. dysenteriae***	**Total** **n= 36(%)**
Trimethoprim-sulphamethoxazole (1.25/23.75 μg)	17(89.4)	9(81.8)	2(50)	1(50)	29(80.5)
Ampicillin(10µg)	14(73.6)	7(63.6)	2(50)	0	23(63.8)
Tetracycline(30µg)	15(78.9)	5(45.4)	0	1(50)	21(58.3)
Nalidixic-acid (30mg)	4(21)	6(54.4)	0	0	10(27.7)
Ceftriaxone (30µg)	0	0	0	0	0
Ciprofloxacin(5µg)	0	0	0	0	0
Chloramphenicol (30µg)	6(31.5)	3(27.2)	2(50)	1(50)	12(33.3)
Gentamicin (10µg)	5(26.3)	7(63.6)	1(25)	0	13(36.1)
Cefixime (5µg)	4(21)	1(9)	1(25)	0	6(16.6)
MDR	14(73.6)	3(27.2)	0	0	17(47.2)

MDR: multidrug resistance

## Results

In the current study, 705 stool samples were collected. During the study period *Shigella* species were isolated from stool specimens of 36/705 (5.1%) pediatric age group, admitted in pediatric ward. The predominant serogroup was *S. flexneri* 19 (52.7%) followed by *S. sonnei* 11(30.5%), *S. boydii* 4 (11.1%), and *S. dysenteriae* 2 (5.5%). Generally 392 (55.6%) of patients were female and 313 (44.4%) of them were male.

The predominant age group of patients who were positive for *Shigella* was between 1 year to 5 years and the least frequent affected age group was less than one year (Table 1). 

The antibiotic resistance patterns and also multidrug resistance rates of* Shigella* isolates was shown in Table 2. In the present study the resistant patterns of 9 different commonly applied antibiotics were as follows: trimethoprim-sulphamethoxazole (80.5%), ampicillin (63.8%), tetracycline (58.3%), gentamycin (36.1%), chloramphenicol (33.3%), nalidixic acid (27.7%), and cefixime (16.6%). All the s*higella* strains were susceptible to ciprofloxacin and ceftriaxone. Of the *Shigella* isolates, 47.2% were resistant to two or more antibiotic. The most common multidrug resistance pattern was to trimethoprim-sulphamethoxazole, ampicillin, and tetracycline. 

## Discussion

Among diarrhoeagenic agents, *Shigella* should be emphasized because of its prevalence and the severity of the associated disease, accounting for 140 million cases globally per year and 60,000 deaths annually of which 60% occur in children below 5 years of age. The geographical distribution, frequency of occurrence and the pathogenicity of the four *Shigella *spp. are different by country and also different among populations within a country (7-10). In endemic regions of the developing countries, shigellosis is predominantly a pediatric disease, with the urban poor being hardest hit. In our present study, the prevalence of shigellosis was 5.1%, which is similar to other studies from other parts of the world, for example; Ghana (11), Dibrugarh, India (12), Cameroon (13), Vellore, and South India (14) that documented rates of 5%, 5.03%, 4.5%, and 5.4%, respectively. Other parts of Iran report dissimilar findings (15,16). Some studies used similar cultural methods and media, and also sampled all age groups reports dissimilar findings, for instance north of Iran (14.05%) (17), and Tehran (1%) (18). 

In this study, we found *S. flexneri* was the most common species, and then followed by *S. sonnei*, which is comparable with studies in Pakistan (19), Kuwait (20), and India (12). In the study from Thailand, *S. sonnei* was the most frequent type, which was isolated from the patients (21). The predominant strains in other regions of Iran differ from our study for example in Shiraz (33) and Tehran (18), the commonest serogroup reported was *S. sonnei* followed by *S. flexeneri*. In another study from Tehran, *S. sonnei* was the most common isolates of *Shigella *(22).

The most frequent age group in this study was age 1-5 years, which was similar to other studies (23, 24). The least frequent affected age group was less than one year. In contrast to our findings, some reports from the United States (36) and Iran (16), indicating a rise in the average age of *Shigella* infection to 24 and ≥12 years respectively. The guiding principle for the select of antibiotic in developing countries comprise the attainability of the drug, worth, and the patterns of resistance in the community (25). Survey of existing data demonstrates a worldwide increase in antimicrobial resistance. Several studies from different parts of the world offered raise in resistance between various strains of *Shigella* against commonly used antibiotic such as trimethoprim-sulphamethoxazole, ampicillin, and tetracycline (26-28). In the present study majority of our *Shigella* isolates were resistant to trimethoprim-sulphamethoxazole (80.5%), ampicillin (63.8%), and tetracycline (58.3%), which is in agreement with observations from Iran, India, Chile, and Nepal (17,29-31). The emergence of resistant to this drugs that utilized as an empirical therapy for treatment of shigellosis may be due to excessive and inappropriate use of them in the study area. Similarly high rates of resistance to the trimethoprim-sulphamethoxazole, ampicillin, and tetracycline of 92.2%, 65.6%, and 65.6%, respectively, were reported in Iran (18). Multidrug-resistant patterns among bacterial pathogens are now common in developing countries (32). In the present study, 47.2% of *Shigella*, isolates was resistant to two or more antibiotic including trimethoprim-sulphamethoxazole, ampicillin, and tetracycline. This is similar to other observations in many parts of the world (11,33-35). Notably, trimethoprim-sulphamethoxazole, ampicillin, and tetracycline had no appropriate role in the empirical treatment of shigellosis in this region. 

In our study, *Shigella* was resistant against cefixime in 0-25% according to different isolates. However, in another study from North of Iran, all isolates were resistant to cefixime (5).

In our study no resistance was found against ciprofloxacin and ceftriaxone. This is similar to results of the studies from Iran (5,15,36) and Kuwait (20) in which all the isolated bacteria showed susceptibility to these antibiotics. However Jain SK et al. (37) and Moez Ardalan K et al. (16) also indicate ceftriaxone and ciprofloxacin have been shown to be highly effective for treatment of shigellosis. On the other hand a recent report from Andaman Islands, India (38) described an increase of resistance among *shigella* isolates to ceftriaxone and ciprofloxacin. 

In conclusion, *S. flexneri* was the predominant species. Hence, we suggest reconsideration of the empiric use of these antimicrobial agents for the treatment of shigellosis. Clinicians should be informed of the vast multidrug resistance rates of *Shigella *spp., especially resistance to trimethoprim-sulphamethoxazole and ampicillin. There was no resistance against ceftriaxone and ciprofloxacin.

## References

[1] Von Seidlein L, Kim DR, Ali M, Lee H, Wang X, Thiem VD (2006). A multicentre study of Shigella diarrhoea in six Asian countries: disease burden, clinical manifestations, and microbiology. PLoS Med.

[2] Jesudason MV, Lalitha MK, Koshi G (1985). Changes in incidence of shigella subgroups and their antibiotic susceptibility pattern in Vellore, South India. J Trop Med Hyg.

[3] Munshi M, Haider K, Rahaman M, Sack D, Ahmed Z, Morshed M (1987). Plasmid-mediated resistance to nalidixic acid in Shigella dysenteriae type 1. Lancet.

[4] Ashkenazi S, May-Zahav M, Sulkes J, Zilberberg R, Samra Z (1995). Increasing antimicrobial resistance of Shigella isolates in Israel during the period 1984 to 1992. Antimicrob Agents Chemother.

[5] Esmaeili Dooki MR, Rajabnia R, Barari Sawadkohi R, Mosaiebnia Gatabi Z, Poornasrollah M, Mirzapour M (2014). Bacterial entropathogens and antimicrobial susceptibility in children with acute diarrhea in Babol, Iran. Caspian J Intern Med.

[6] Pourakbari B, Mamishi S, Mashoori N, Mahboobi N, Ashtiani MH, Afsharpaiman S (2010). Frequency and antimicrobial susceptibility of Shigella species isolated in Children Medical Center Hospital, Tehran, Iran, 2001-2006. Braz J Infect Dis.

[7] Green MS, Block C, Cohen D, Slater PE (1991). Four decades of shigellosis in Israel: epidemiology of a growing public health problem. Rev Infect Dis.

[8] Zaman K, Yunus M, Baqui A, Hossain K (1991). Surveillance of shigellosis in rural Bangladesh: a 10 years review. J Pak Med Assoc.

[9] Al-eissa Y, Al-zamil F, Al-kharashi M, Kambal A, Chowdhury M, Al-ayed I (1992). The relative importance of Shigella in the aetiology of childhood gastroenteritis in Saudi Arabia. Scand J Infect Dis.

[10] Ergönül O, Imre A, Celikbaş A, Dokuzoğuz (2004). Drug resistance of Shigella species: changes over 20 years in Turkey. Int J Antimicrob Agents.

[11] Opintan J, Newman MJ (2007). Distribution of serogroups and serotypes of multiple drug resistant Shigella isolates. Ghana Med J.

[12] Nath R, Saikia L, Choudhury G, Sharma D (2013). Drug resistant Shigella flexneri in & around Dibrugarh, north-east India. Indian J Med Res.

[13] Njunda AL, Assob JC, Nsagha DS, Kamga HL, Awafong MP, Weledji EP (2012). Epidemiological, clinical features and susceptibility pattern of shigellosis in the Buea Health District, Cameroon. BMC Res Notes.

[14] Jesudason MV (2002). Shigella isolation in Vellore, South India (1997-2001). Indian J Med Res.

[15] Jafari F, Hamidian M, Salmanzadeh-Ahrabi S, Bolfion M, Kharaziha P, Yaghobi M (2008). Molecular diagnosis and antimicrobial resistance pattern of Shigella spp isolated from patients with acute diarrhea in Tehran, Iran.. Gastroenterol Hepatol Bed Bench.

[16] MoezArdalan K, Zali MR, Dallal MM, Hemami MR, Salmanzadeh-Ahrabi S (2003). Prevalence and pattern of antimicrobial resistance of Shigella species among patients with acute diarrhoea in Karaj, Tehran, Iran. J Health Popul Nutr.

[17] Savadkoohi RB, Ahmadpour-Kacho M (2007). Prevalence of Shigella species and their antimicrobial resistance patterns at Amirkola children hospital, North of Iran. Iran J Pediatr.

[18] Mardaneh J, Poor SA, Afrugh P (2013). Prevalence of Shigella species and Antimicrobial Resistance Patterns of Isolated Strains from Infected Pediatrics in Tehran. Int J Enterpathog.

[19] Zafar A, Sabir N, Bhutta ZA (2005). Frequency of isolation of Shigella serogroups/serotypes and their antimicrobial susceptibility pattern in children from slum areas in Karachi. J Pak Med Assoc.

[20] Jamal WY, Rotimi VO, Chugh TD, Pal T (1998). Prevalence and susceptibility of Shigella species to 11 antibiotics in a Kuwait teaching hospital. J Chemother.

[21] Pulsrikarn C, Bangtrakulnonth A, Pornruangwong S, Sriyapai T, Sawanpanyalert P, Aswapokee N (2009). Shigella species and serotypes among clinical isolates in Thailand from 2001 to 2005. J Med Assoc Thai.

[22] Tajbakhsh M, Garcia Migura L, Rahbar M, Svendsen CA, Mohammadzadeh M, Zali MR (2012). Antimicrobial-resistant Shigella infections from Iran: an overlooked problem?. Antimicrob Agents Chemother.

[23] Rolfo F, Marin GH, Silberman M, Pattin J, Giugnio S, Gatti B (2011). Epidemiological study of shigellosis in an urban area of Argentina. J Infect Dev Ctries.

[24] Sousa MÂ, Mendes EN, Collares GB, Péret-Filho LA, Penna FJ, Magalhães PP (2013). Shigella in Brazilian children with acute diarrhoea: prevalence, antimicrobial resistance and virulence genes. Mem Inst Oswaldo Cruz..

[25] DeRoeck D, Clemens JD, Nyamete A, Mahoney RT (2005). Policymakers’ views regarding the introduction of new-generation vaccines against typhoid fever, shigellosis and cholera in Asia. Vaccine.

[26] Ahmed K, Shakoori FR, Shakoori AR (2003). Aetiology of shigellosis in northern Pakistan. J Health Popul Nutr.

[27] Hossain MA, Rahman M, Ahmed Q, Malek M, Sack R, Albert M (1998). Increasing frequency of mecillinam-resistant Shigella isolates in urban Dhaka and rural Matlab, Bangladesh: a 6 year observation. J Antimicrob Chemother.

[28] Ahmed AA, Osman H, Mansour AM, Musa HA, Ahmed AB, Karrar Z (2000). Antimicrobial agent resistance in bacterial isolates from patients with diarrhea and urinary tract infection in the Sudan. Am J Trop Med Hyg.

[29] Fulla N, Prado V, Duran C, Lagos R, Levine MM (2005). Surveillance for antimicrobial resistance profiles among Shigella species isolated from a semirural community in the northern administrative area of Santiago, Chile. Am J Trop Med Hyg.

[30] Pazhani G, Ramamurthy T, Mitra U, Bhattacharya S, Niyogi S (2005). Species diversity and antimicrobial resistance of Shigella spp. isolated between 2001 and 2004 from hospitalized children with diarrhoea in Kolkata (Calcutta), India. Epidemiol Infect.

[31] Alici O, Açikgöz Z, Gamberzade S, Göcer S, Karahocagil M (2006). Antibiotic resistance rates of Shigella species isolated from stool cultures in the years 1999-2003]. Mikrobiyol Bul.

[32] Tjaniadi P, Lesmana M, Subekti D, Machpud N, Komalarini S, Santoso W (2003). Antimicrobial resistance of bacterial pathogens associated with diarrheal patients in Indonesia. Am J Trop Med Hyg.

[33] Lee JC, Oh JY, Kim KS, Jeong YW, Cho JW, Park JC (2001). Antimicrobial resistance of Shigella sonnei in Korea during the last two decades. APMIS.

[34] Jeong YS, Lee JC, Kang HY, Yu HS, Lee EY, Choi CH (2003). Epidemiology of nalidixic acid resistance and TEM-1-and TEM-52-mediated ampicillin resistance of Shigella sonnei isolates obtained in Korea between 1980 and 2000. Antimicrob Agents Chemother.

[35] Badalian K, Tavakoli H (1976). Transfer of drug resistance factor in Shigella sonnei isolated in Iran. Pahlavi Med J.

[36] Qureishi M, Borji A, Bokaeian M, Roudbari M, Shahraki S, Niazi A (2008). Antimicrobial resistance of Shigella spp isolated from diarrheal patients in Zahedan.. Acta Med Iranica.

[37] Jain SK, Gupta A, Glanz B, Dick J, Siberry GK (2005). Antimicrobial-resistant Shigella sonnei: limited antimicrobial treatment options for children and challenges of interpreting in vitro azithromycin susceptibility. Pediatr Infect Dis J.

[38] Bhattacharya D, Sugunan A, Bhattacharjee H, Thamizhmani R, Sayi D, Thanasekaran K (2012). Antimicrobial resistance in Shigella-rapid increase & widening of spectrum in Andaman Islands, India. Indian J Med Res.

